# Electrophysiological Biomarkers of Epileptogenicity in Alzheimer’s Disease

**DOI:** 10.3389/fnhum.2021.747077

**Published:** 2021-11-30

**Authors:** Tingting Yu, Xiao Liu, Jianping Wu, Qun Wang

**Affiliations:** ^1^Department of Neurology, Beijing Tiantan Hospital, Capital Medical University, Beijing, China; ^2^China National Clinical Research Center for Neurological Diseases, Beijing, China; ^3^Advanced Innovation Center for Human Brain Protection, Capital Medical University, Beijing, China; ^4^Collaborative Innovation Center for Brain Disorders, Beijing Institute of Brain Disorders, Capital Medical University, Beijing, China

**Keywords:** Alzheimer’s disease, epileptogenesis, seizure, electrophysiology, biomarkers

## Abstract

Cortical network hyperexcitability is an inextricable feature of Alzheimer’s disease (AD) that also might accelerate its progression. Seizures are reported in 10–22% of patients with AD, and subclinical epileptiform abnormalities have been identified in 21–42% of patients with AD without seizures. Accurate identification of hyperexcitability and appropriate intervention to slow the compromise of cognitive functions of AD might open up a new approach to treatment. Based on the results of several studies, epileptiform discharges, especially those with specific features (including high frequency, robust morphology, right temporal location, and occurrence during awake or rapid eye movement states), frequent small sharp spikes (SSSs), temporal intermittent rhythmic delta activities (TIRDAs), and paroxysmal slow wave events (PSWEs) recorded in long-term scalp electroencephalogram (EEG) provide sufficient sensitivity and specificity in detecting cortical network hyperexcitability and epileptogenicity of AD. In addition, magnetoencephalogram (MEG), foramen ovale (FO) electrodes, and computational approaches help to find subclinical seizures that are invisible on scalp EEGs. We performed a comprehensive analysis of the aforementioned electrophysiological biomarkers of AD-related seizures.

## Introduction

Brain rhythms are fundamental in maintaining normal cognition and behavior, and neuronal hyperexcitability has emerged as an important electrical abnormality that could not only lead to memory failure in the early stage of Alzheimer’s disease (AD) but also contribute to the progression of the disease (Noebels, 2011; Vossel et al., 2013; Busche and Konnerth, 2015, 2016; Palop and Mucke, 2016). Once neuronal hyperexcitability of the cerebral cortex is established, it can manifest as epileptic seizures or subclinical epileptiform discharges. Seizures are reported in 10–22% of patients with AD (Friedman et al., 2011; Vossel et al., 2013; Cretin et al., 2016; Sarkis et al., 2016), and subclinical epileptiform activities have been found in more than 40% of patients with AD in a recent prospective electroencephalogram (EEG) study (Vossel et al., 2016).

The presence of seizure and subclinical epileptiform activities have been shown to contribute to impaired memory and attention, especially cognitive fluctuation (Palop and Mucke, 2009; Vossel et al., 2016). A greater extent of neuronal hyperactivity tends to occur in the earliest stages of AD compared with later stages, which has been shown from both fMRI studies in humans and neuronal activity studies in mouse models of AD (Zott et al., 2018). “Antiepileptogenic” therapies in AD appear to be feasible in order to delay the progression of the disease. Although they are easy to implement, performing these treatments in all patients with AD presents a problem as the majority of patients with AD would not have any benefit, though the subset of patients with AD who have epileptiform activity might benefit greatly from early treatment with antiepileptic drugs (AEDs). Hence, effective treatment first requires the detection and suppression of seizures and subclinical epileptiform activity. In this study, we performed a comprehensive analysis of the potential electrophysiological biomarkers of AD-related seizures in humans and discuss their feasibility in clinical practice and their potential to predict the subsequent development of clinical seizures and epilepsy.

## Scalp Electroencephalogram

### Epileptiform Discharges

**FIGURE 1:**
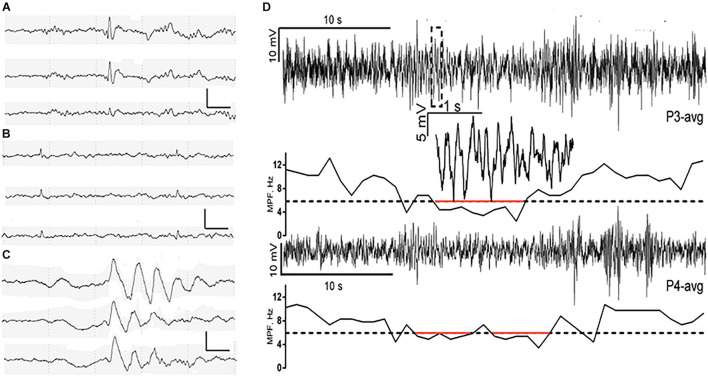
Characterization of spike, small sharp spike (SSS), temporal intermittent rhythmic delta activities (TIRDA), and paroxysmal slow-wave event (PSWE). **(A–C)** Representative examples of spike, SSS, and TIRDA from the right temporal region in three patients with AD from our own ward, respectively. Scalp electrodes were placed using the international 10–20 system, sampling at 200 Hz. EEG channels (top to bottom): F8-ave, T4-ave, and T6-ave. Calibration bars: 100 μV and 500 ms. **(D)** A PSWE detected in a patient with AD. Traces from electrodes P3 (upper trace) and P4 (averaged as reference) are shown. The segment within the dashed rectangle of P3 is shown magnified. The median power frequency is presented below each trace. Segments below 6 Hz (dashed line) are marked in red. From Milikovsky et al. (2019). Reprint with permission from The American Association for the Advancement of Science (AAAS).

The best-known biomarker of hyperexcitability in humans is the epileptiform discharge, which is defined as a paroxysmal EEG graphoelement (spike or sharp wave) with a duration of 20–200 ms that is clearly distinct from ongoing background EEG activity followed by slow waves (Noachtar and Remi, 2009) (Figure 1A). On scalp EEGs, visible subclinical epileptiform discharges occur in 9–21% of patients with AD who had no prior history of epilepsy or seizure, a higher rate than 0–5% of healthy controls (Vossel et al., 2016; Brunetti et al., 2020; Lam et al., 2020). Long-term EEG detection has found that epileptiform discharges in patients with AD mainly occur during a sleep state and are largely lateralized to the temporal lobe, especially the left temporal lobe.

Epileptiform discharges that had more robust morphological features including significantly larger trough voltage, peak-to-trough voltage, and slope of falling half-wave of the peak are more strongly associated with clinical seizures in AD (Lam et al., 2020). The guiding significance of robust epileptiform discharges is similar to giant spikes (simultaneous spikes in all channels, with massive voltages > ± 10 SD from the filtered baseline in at least one channel) observed in transgenic mice with APP/PS1 mutations (Gureviciene et al., 2019). The same indicative effect being strongly associated with clinical seizures has also been seen in right temporal epileptiform discharges (100% specificity and 43% sensitivity) (Lam et al., 2020).

The detection rate of epileptic discharges in patients with AD by scalp EEG is significantly correlated with recording time. Approximately, 62% of patients with AD showed epileptic discharges in EEG recordings lasting 24 h (Horvath et al., 2018) and only 3% in 20 min of eyes-closed EEG recordings (Liedorp et al., 2010). Most epileptiform discharges from patients with AD occur during sleep, requiring overnight EEG monitoring for detection. Epileptiform discharges occurred most frequently during N2 sleep, while awake and rapid eye moment (REM) states are the least permissive states for the expression of epileptiform discharges in patients with focal epilepsy (Sammaritano et al., 1991; Malow et al., 1997; Diaz-Negrillo, 2013), and the results of EEG monitoring in patients with AD are consistent with this. While epileptiform discharges detected during awake or REM states were suggestive for clinical seizures (85.7% specificity and 85.7% sensitivity) (Lam et al., 2020), the study by Lam et al. (2020) also pointed out that patients with AD who had high frequencies of epileptiform discharges were more likely to have generalized convulsions.

Lam et al. (2017a) found that spikes (sharply contoured transients, clearly distinguishable from, and usually interrupting background activities, with a duration of <70 ms) from patients with aMCI were largely lateralized to the left mesial temporal lobe (mTL), whereas they were largely lateralized to the right mTL in moderate patients with AD. Thus, they proposed that there might be left hemisphere hyperactivity predisposition and left mTL susceptibility in the early stages of AD. Data from anatomical and functional connectivity modalities also support this; the result may also be mediated in part by the E4 variant of apolipoprotein E (APOE4) allele (Thompson et al., 2003; Damoiseaux et al., 2009; Donix et al., 2013; Wessa et al., 2016; Yang et al., 2017; Liu et al., 2018; Zott et al., 2018).

Studies in animal models with AD have established a direct link between neuronal hyperactivity and propagation of amyloid and tau pathology (Cirrito et al., 2005; Busche et al., 2012; Pooler et al., 2013; Wu et al., 2016). Reyes-Marin and Nunez (2017) also found that the frequency of epileptiform-like discharges was significantly correlated with the number of amyloid-β plaques in APP/PS1 mice. The results of these animal studies illustrate that the asymmetry in temporal lobe hyperexcitability might be related to an asymmetric cascade of AD pathology.

Finally, there is growing evidence that scalp EEG epileptiform discharges in patients with AD have a variable association with clinical seizures (Vossel et al., 2016; Lam et al., 2020). With an infrequent frequency (<10 per 24 h), these epileptiform discharges typically arise locally from the lateral temporal cortex or propagate to the surface from deep mTL foci (Bakker et al., 2012; Lam et al., 2017a). Thus, to some extent, scalp EEG epileptiform discharges alone are limited, and they may not be the optimal biomarkers for epileptogenicity in AD.

### Small Sharp Spikes

Small sharp spikes (SSSs), also known as benign sporadic sleep spikes (White et al., 1977), are low amplitude (30–50 uV), short duration (<50 ms) spikes that occur during early drowsiness, and N1 and N2 sleep stages (Figure 1B). They are widely distributed in bilateral brain hemispheres typically seen independently over the bi-frontotemporal regions but can also occur unilaterally. In addition, SSSs are usually considered as a benign variant of EEG, which has no association with epilepsy. However, recent studies have indicated that SSS-like waveforms in the scalp might be related to mTL epileptiform discharges, especially unilateral SSS-like waveforms (Lam et al., 2017a; Issa et al., 2018). According to the study by Lam et al. (2020) unilateral (left or right temporal region) SSS-like waveforms with high frequency (>100 per 24 h) are associated with clinical seizure in AD. However, pinpointing SSSs as an electrophysiological biomarker of seizure in patients with AD requires further validation in more well-designed studies to distinguish pathological SSSs from benign SSSs.

### Temporal Intermittent Rhythmic Delta Activity

Temporal intermittent rhythmic delta activity (TIRDA) refers to the delta activity distributed in the temporal lobe with a frequency of 2.5–3.0 Hz and a sinusoidal or serrated waveform that occurs repeatedly and intermittently and has a strong association with mesial temporal lobe epilepsy (mTLE) (Reiher et al., 1989; Gambardella et al., 1995; Normand et al., 1995; Gennaro et al., 2003) (Figure 1C). TIRDA occurs in 26% of patients with AD-related epilepsy (Lam et al., 2020). Although occurring at a lower frequency in patients with AD without epilepsy, TIRDA has been associated with higher diagnostic confidence compared to epileptiform discharges (positive predictive value for determining epileptiform, 83.3 vs. 61.5%) (Lam et al., 2020). Like epileptiform discharges, TIRDA is more likely to occur during N2 sleep and in the left temporal location, while TIRDA that does occur during awake or REM states is more strongly associated with clinical seizures in AD (Lam et al., 2020). What is more, the lateralization of TIRDA matches that of epileptiform discharges in patients with AD. However, because of its low frequency (<10 per 24 h) of occurrence in AD, the utility of TIRDA as a quantitative biomarker is somewhat limited (Lam et al., 2020).

### Paroxysmal Slow Cortical Activity

Slowing of scalp EEG activity has been observed in AD by studying early quantitative EEG power changes in patients with AD (Musaeus et al., 2018), and the EEG slowing correlates with decreased cognition in patients with AD and unimpaired older adults (Stomrud et al., 2010; Musaeus et al., 2018). The EEG slowing might be a potential sign of neural network dysfunction. Milikovsky et al. (2019) analyzed the temporal characteristics of EEG slowing from patients with AD and found that cortical slowing is in part composed of transient paroxysmal slowing of the cortical network. This transient paroxysmal slowing of the cortical network is called paroxysmal slow wave events (PSWEs), which refer to “events” when the median power frequency is lower than 6 Hz for at least 5 consecutive s on the scalp EEG (Milikovsky et al., 2019) (Figure 1D). These PSWEs were also obtained in patients with epilepsy, animal models of AD, and animal models of epilepsy.

Investigating PSWE characteristics in aged mice, young 5xFAD mice, and young rats with epilepsy, Milikovsky et al. (2019) found that there was a spatial correlation between PSWEs and blood-brain barrier dysfunction (BBBd). They also observed a causal link between BBBd and PSWE in animal models by infusing albumin into the lateral ventricle of rats and then detecting the PSWEs in epidural recordings after a month of infusion. Their results indicate that PSWEs can be an indicator for the subclinical seizure-like activity that reflects microvascular pathology. Hence, PSWE detection in routine scalp EEG recordings might be an affordable and sensitive diagnostic indicator for subclinical seizures among patients with AD and as a pharmacodynamic measure for AEDs. However, we must realize that seizure and epileptiform activity mostly occur in the mTL in patients with AD and are often undetectable by scalp EEG recording. Thus, more effective approaches that can detect epileptiform discharges in the mTL are needed to improve the efficiency of scalp EEG monitoring.

## Magnetoencephalography

Magnetoencephalography (MEG), which is thought to be more sensitive to discharges of tangential sulcal sources than EEG with a high temporal and spatial resolution (Oishi et al., 2002), has also been widely used as a non-invasive tool to assess mTL activity and localized epileptogenic lesions in an epilepsy non-invasive tool (Enatsu et al., 2008; Kaiboriboon et al., 2010; Wennberg et al., 2011; Wennberg and Cheyne, 2014). Although the main application field of MEG is the presurgical evaluation of drug-resistant epilepsy, MEG also provides a complementary approach to scalp EEG in detecting cortical network dysfunction in patients with AD. Vossel et al. (2016) prospectively assessed the epileptiform activities in 33 patients with AD and 19 age-matched healthy controls, by 1-h resting MEG recordings and overnight scalp EEG recordings. Vossel et al. (2016) found that visible epileptiform discharges on MEG occurred in 33.3% of patients with AD, much higher than that of 21.1% on overnight scalp EEGs. Although the specificity of MEG might not be as high as that of overnight EEG (11% of healthy controls had epileptiform discharges visible on MEG while had them on overnight EEG), patients with epileptiform activity on MEG or overnight EEG had significantly faster rates of cognitive decline than those without epileptiform activity (Vossel et al., 2016). These findings suggest that MEG is an effective electrophysiological biomarker of cortical hyperexcitability in patients with AD with much higher sensitivity than scalp EEG. Interestingly, epileptiform discharges detected by MEG were more right-sided compared with the more left-sided epileptiform discharges detected by scalp-EEG (Vossel et al., 2016). Different detection capabilities or different interpretation techniques may contribute to the discordance, but the principle behind this remains unclear.

Importantly, epileptiform discharges in patients with AD mainly occur during a sleep state, while the MEG recording cannot exceed a few hours at a time. Thus, it is nearly impossible to capture natural sleep or paroxysmal events during a sleep state by MEG. Considering the high requirement of equipment maintenance and the high costs of MEG examination, it is difficult to carry out MEG as a routine examination in general patients with AD. However, for patients with high suspicion of cortical hyperexcitability without visible epileptiform discharge on scalp EEG, MEG examination can be chosen to individually guide the usage of AEDs.

## Foramen Ovale Electrodes

Foramen ovale (FO) electrodes are a semi-invasive alternative to stereo-EEG electrodes. After general endotracheal anesthesia is induced, a single multi-contact FO electrode is positioned adjacent to each mTL inserted through the cheek skin to the ipsilateral natural aperture (FO) in the skull (Sheth et al., 2014). High-quality, long-term recordings can be obtained directly from the mTL using FO electrodes (Sperling et al., 1986; Fernández Torre et al., 1999). As FO electrodes will cause no skull defect, scalp EEG recording can be carried out simultaneously with FO electrodes. Furthermore, as mentioned above, since the mTL is the most affected location in patients with AD and FO electrodes that are accurate in capturing samples from deep temporal activities might be the best method for assessing subclinical discharges in patients with AD (Lam et al., 2019).

Lam et al. (2017a) used bilateral FO electrode recordings in two patients with AD (one had aMCI and another had moderate dementia; both had cerebrospinal fluid biomarker-confirmed AD) for the first time. The FO electrode recordings of both patients with AD demonstrated abundant, sleep-activated (especially non-REM sleep) spikes, and over 95% of the spikes were invisible on the simultaneous scalp EEG. In addition, three silent mTL seizures were captured on FO electrode recordings, while no visible evidence was found on scalp EEG in the patient with aMCI. They also found that mTL spikes on FO electrode recordings in the patient with aMCI occurred at up to a 10-fold rate compared with the patient with moderate dementia, supporting the concept that a greater extent of neuronal hyperactivity had developed during the earliest stages of AD rather than later stages (Zott et al., 2018). Existing studies indicate that mTL epileptiform discharges detected by FO electrodes mostly occur during a sleep state in both humans and mouse AD models (Lam et al., 2017a; Brown et al., 2018) but non-REM sleep in humans (Lam et al., 2017a; Brown et al., 2018) and REM sleep in mouse models with AD (Kam et al., 2016; Brown et al., 2018). Although FO electrodes offer high quality and long-term recordings of mTL activity, their utility as a screening tool for epileptiform discharges in patients with AD is limited because of their high cost, potential risks (for example, central nervous system infection and bleeding), and the requirement of good neurosurgical skills for electrode placement.

## Computational Approaches

For patients with AD, routine scalp EEG monitoring (20–40 min) is both necessary and feasible for the purpose of observing brain rhythms, but it is less helpful in recording epileptiform activity (Liedorp et al., 2010). However, performing overnight EEG, MEG, or FO electrode recordings is not feasible for the reasons of hospital resources, willingness to monitor patients, and high costs. As such, scientists have developed non-invasive and inexpensive methods, which can be widely implemented for diagnosing epileptiform activity.

Many studies have demonstrated that though there is a lack of visible epileptiform activity on scalp EEG, non-specific subtle and quantitative changes in local or long-distance networks induced by mTL spikes or seizures (Tyvaert et al., 2009; Vulliemoz et al., 2011; Cunningham et al., 2012; Aghakhani et al., 2015; Khoo et al., 2017; Naftulin et al., 2018) can be detected indirectly using artificial intelligence approaches. In a proof-of-principle study, Lam et al. (2016) analyzed the EEG data of 25 patients with epilepsy who underwent scalp EEG recording and FO electrode recording. By dividing scalp EEG recording into epochs and extracting coherence features of each epoch, they trained a seizure detector which correctly identified 40% of scalp-negative seizures (seizures detected by FO electrode recording but invisible on the scalp EEG), with a positive predictive value of 75% (Lam et al., 2016). In another publication by the same authors (Lam et al., 2017b), they extracted scalp EEG spectral power as an input feature and trained a machine learning algorithm that correctly identified 50% of scalp-negative seizures, with a positive predictive value of 94%. Studies by other researchers (Nayak et al., 2004; Koessler et al., 2015) also pointed out that mTL spikes or seizures might be detected by quantitative EEG signatures though they are invisible on a scalp EEG. Babiloni et al. (2020) found that higher temporal delta source activities are more strongly associated with clinical seizures in AD by estimating regional EEG cortical sources using exact low-resolution brain electromagnetic tomography (eLORETA) freeware. They used the area under receiver operating characteristic (AUROC) curves to index the accuracy of eLORETA solutions in identifying seizures and found an accuracy of 69% (Babiloni et al., 2020).

Development of computational approaches that accurately identify spikes or seizures from scalp EEG or MEG is underway (Lam et al., 2016, 2017b; Spyrou et al., 2016; Pizzo et al., 2019). However, this might be difficult to validate with large clinical data sets of combined scalp EEG/MEG and intracranial (especially FO electrode) recordings. Once the computational approaches become established, patients with AD may obtain more accurate guidance on antiepileptic therapy.

## Discussion

Patients with AD have an increased risk of seizures, compared to an age-matched control population, and they are over 10 times more likely to develop epilepsy, especially patients with early onset familial AD (Amatniek et al., 2006; Pandis and Scarmesa, 2012). The significance of interictal epileptiform activities in patients with AD is still controversial, and they are biomarkers of hyperexcitability, but their relation to seizures is actually unknown. Interictal epileptiform activities may compromise memory formation and consolidate themselves, even in the absence of seizures. There are also studies that contend that seizures might be a critical part of AD pathogenesis (Scarmeas et al., 2009). Numerous studies (Reyes-Marin and Nunez, 2017; Gureviciene et al., 2019) in animal models also suggest the importance of detecting these events, finding that a high number of interictal spikes increases the risk of seizures.

However, one thing most studies agree on is that patients with AD with seizures or subclinical epileptiform activity experience faster cognitive declines over time (Vossel et al., 2016), and patients with AD might benefit from antiepileptic therapy. Moreover, the identification of biomarkers of epileptogenesis in patients with AD is a prerequisite for designing and developing targeted therapeutic approaches.

## Conclusion

To date, human studies have identified several candidate EEG biomarkers as follows: epileptiform discharges (especially those with specific features, including high frequency, robust morphology, right temporal location, and occurrence during awake or REM states), frequent SSSs, TIRDA, and PSWEs recorded by scalp EEG (Table 1). In addition, MEG, FO electrodes, and computational approaches help to find subclinical seizures that are invisible on scalp EEG electrodes in patients with AD (Table 1). However, these findings require further study in humans as well as in animals to validate them and determine which are reliable and feasible electrophysiological biomarkers of epileptogenicity in patients with AD.

**TABLE 1:** Electrophysiological biomarkers of AD-related increase of cortical excitability.

	**Characteristics associated with clinical seizure**	**Advantages**	**Disadvantages**	
Scalp EEG		Non-invasive		
Epileptiform discharges	Robust morphology, right temporal location, occur during awake or REM state, and higher frequency	Simple to implement in clinical work	Required long recording time and insensitive to epileptic discharges from deep mTL foci	Liedorp et al., 2010; Bakker et al., 2012; Vossel et al., 2016; Lam et al., 2017a, 2020; Horvath et al., 2018
SSSs	Unilateral SSS-like waveforms and frequent frequency (>100 per 24 h)	Simple to implement in clinical work	Hard to distinguish pathologic from benign SSSs	Lam et al., 2017a, 2020; Issa et al., 2018
TIRDA	Occur during awake or REM state	High diagnostic confidence	Infrequently frequency (<10 per 24 h)	Lam et al., 2020
PSWEs	–	No requirement for long recording time	Hard to distinguish	Milikovsky et al., 2019
MEG	–	Non-invasive and sensitive to discharges of tangential sulcal sources	Low specificity, high requirement of equipment, and only be monitored for a short time at a time	Oishi et al., 2002; Vossel et al., 2016
FO electrodes	Occur during non-REM sleep state	High quality, long-term recording, capture samples from deep temporal activities, and no skull defect	High cost, semi-invasive, and request for good neurosurgical skills of electrode placement	Sheth et al., 2014; Lam et al., 2017a, 2019
Computational approaches	–	Non-invasive and short monitoring time	Request for validation with large clinical data sets	Lam et al., 2016, 2017b; Babiloni et al., 2020

*AD, Alzheimer’s disease; SSS, small sharp spikes; TIRDA, temporal inter mitten rhythmic delta activity; PSWEs, paroxysmal slow wave events; EEG, electroencephalogram; MEG, magnetoencephalogram; FO, foramen ovale; mTL, mesial temporal lobe; REM, rapid eye moment.*

## Author Contributions

TY chose the search term, determined the methodology of the review, and drafted the manuscript. XL helped to choose the search term and determined the methodology of the review. JW and QW revised the manuscript and commented on previous versions of the manuscript. All authors read and approved the final manuscript.

## Conflict of Interest

The authors declare that the research was conducted in the absence of any commercial or financial relationships that could be construed as a potential conflict of interest.

## Publisher’s Note

All claims expressed in this article are solely those of the authors and do not necessarily represent those of their affiliated organizations, or those of the publisher, the editors and the reviewers. Any product that may be evaluated in this article, or claim that may be made by its manufacturer, is not guaranteed or endorsed by the publisher.

## Abbreviations

5xFAD: 5x familial AD
AD: Alzheimer’s disease
AEDs: antiepileptic drugs
aMCI: amnesic mild cognitive impairment
APOE4: the E4 variant of apolipoprotein E
AUROC: area under receiver operating characteristic
BBBd: blood-brain barrier dysfunction
EEG: electroencephalogram
eLORETA: exact low-resolution brain electromagnetic tomography
FO: foramen ovale
MEG: magnetoencephalogram
mTL: mesial temporal lobe
PSWEs: paroxysmal slow wave events
REM: rapid eye moment
SSS: small sharp spikes
TIRDA: temporal inter mitten rhythmic delta activity.
